# Alternation a Law of Vital Action

**Published:** 1875-07

**Authors:** L. C. Ingersoll

**Affiliations:** Keokuk, Iowa


### DENTAL REGISTER.

VOL. XXIX.] JULY, 1875. [No. 7.

### ALTERNATION A LAW OF VITAL ACTION.

BY L. C. INGERSOLL, KEOKUK, IOWA.

*Read before the Illinois State Dental Society, May 12th, and Iowa State Dental Society, May 19th, 1875.*

Vitality is that mysterious something that distinguishes organic from inorganic bodies. We call it vitality, vital force, vital element, vital principle, the principle of animation, the living principle, life. We call it energizing force, a power.—Wheather we define it by one or by many words, neither one nor all are adequate to bring out from the pale of mystery that which we attempt to define, and place it in the clear light of intellectual perception. It is mysterious still, and incomprehensible as mind. Its incomprehensibility forestalls our efforts to gain a conception of its true nature, and renders unphilosophical any attempt at complete definition. Yet we have no difficulty with *ourselves* in this regard when we talk on the subject, for the conscious possession of life as a vitalizing force within us compels the most ready intellectual assent to the fact, inexplicable as it is. When a man says by the fore of inward consciousness, *I live*, he cares not who says, "You know not what you are talking about"—and he scorns any attempt at an analytic solution of the fact. An appeal to consciousness is a sufficient verification of it.

This energizing force of which we are so consciously possessed, is located in and acts through the nervous system in the same mysterious manner that mind—the power of thought and all mental phenomena—is located in and acts through the brain.

The nervous function is peculiar among bodily functions. It does not act physically or chemically as do various functions of the body. Nor is its functional power so localized that it is wholly exerted within and upon itself, like the functions of the stomach, the heart or the transformation power of the tissues. The functions of the nervous system are wholly unlike any known phenomena of other bodily functions. Its power is distributed everywhere, to all organs of the body and presides over and controls their functional activity. The nervous system makes of the great diversity of bodily organs, a harmonious whole. It causes every organ to act in harmony with every other organ, and creates that sympathetic relation between organs remote from each other with widely different functions, which is apparent when disease rudely invades the citadel of life, or any one function becomes unduly stimulated. When an unusual muscular effort is put forth, the circulation of the blood is quickened, and by this mysterious harmony of functional action an increased vital energy is exhibited in the lungs. Were it not so the increased amount of blood carried to the lungs would not be sufficiently oxygenated and the carbonic acid remaining in the blood would soon create disorder.

It being made evident from general observation and carefully conducted experiments, that the exciting cause of all functional action whether in the development and growth of the organ themselves, or directed to maintenance of function or metamorphosis of tissue, resides in and acts through the nervons system as the vitalizing power of the body, it becomes an important study to observe its limitations, and the characteristics of its working.

The most observable charactèristic of vitality is its limited duration. It has different periods for sustaining functional action in different species of organized beings, and in different individuals of the same species. In all organisms it sooner or later becomes extinct, and their devitalized material forms are resolved into their original elements. This we call death. Again, vitality differs in force or strength in different individuals, and apparantly in the same individual at different times.

Another proposition co-ordinate with the one first named, is that vitality is a limited force in the sense of not being adequate to resist every and all influences that oppose vitality. It is, therefore, limited and variable in its power to promote functional action equally in all organs at the same time. I consider this a great physiological fact on which hinge many of the phenomena of development, the performance of function, and well defined pathological conditions.

Mark well the distinction between the fact previously mentioned, that there is an apparent difference in the amount and force of vitality in the same individual at different times, and the fact last mentioned, that there is a limit to its power that functional action is not and can not be promoted at any time, equally in all organs. The former is an indication or symptom of impaired function. The latter is compatible with the most complete functional performance; and, as I propose to show, is to be recognized as a physiological law. Should we find it to be such a law, and not a mere accident of disease or an idiosyncrasy, it will afford a ready explanation of much that is obscure in ætiology, and give an open door to diagnosis through which we may see and account for many cases of observed want of harmony in development, faultiness in structure, and functional weakness.

This leads me to the postulate which I have announced as the subject of this essev,—that *alternation is a law of vital action.*

That vitality is variable in its manifestations when the body is diseased, is a fact of the commonest observation; and the diseased condition is looked to as accounting for any changed manifestation of vital phenomena. But if in the most healthful physiological condition the same variableness and change in vital phenomena appears, it certainly can not be the accident of disease.

The external manifestations by which we in general determine the degree of vitality supporting the organism, is no certain indication of the real amount of vital force mysteriously pervading the whole system. Hence it may not be true that at different times one is possessed of different degrees or different amounts of vital energy—only apparently so.

The fact that in our most hoathful condition vital activity is not equal in all parts, and in all functions of the organism at the same time, compels the conclusion that vitality is limited as an energizing force. If its capabiltties as an exciting force were unlimited, we could see no reason why the same degree of vital activity should not be found in all bodily functions at the same time, promoting a uniform and steady development of all parts of the system equally. But vitality being limited, and physical develepment and growth being unequal, as is plainly evident from well observed facts, the conclusion is inevitable, that vitality as an energizing force alternates in its actions upon different organs according as this one or that needs the vital stimulus to increased action in promoting harmony of development or equi-balance of function. This to my mind admits of plainest proof, and affords explanation of many obscure teachings in practical science.

We will attend first to the proofs, then to the practical application.

The harmonies of nature are so marked and wonderful, that processes which seem intricate in one department, are revealed to our full comprehension by analogous processes in some other department with which we are more familiar.

If we turn our attention to a healthy growing shrub in early spring time, and carefully turn over the earth at its base, we may discover that the root has already made a growth of several inches while there is scarcely a sign of functional activity of the shrub elsewhere observable. If after a few weeks we look at it again we shall see that the leaf-buds or flower-buds have expanded largely, while the root has made very little if any progress beyond the point where we observed it before. The buds unfold, and the leaves expand in size, while there is no apparent growth of stem. Again we look at it and find no perceptible change in the foliage, but we find a growth of wood. At length the stem ceases for a while to grow, no new leaves are being put forth, while the vital forces are occupied in the production of roots to preserve the harmony of development and to secure nutrient support corresponding with the increased and increasing demand. When the flower of a fruit bearing tree drops its petals, the vital forces are directed to the rapid production of fruit; and if the fruit is very abundant the woody fiber ceases almost entirely to grow. The stoned fruits, as the peach and the plumb, always surprise us with their rapid growth in size for the first few weeks, and equally surprise us in the succeeding weeks by apparently ceasing to grow at all. There is no increase of the young fruit in size, but nature is occupying her forces in hardening and maturing the stone, and the enclosed seed germ.

This example from one of the great kingdoms of organic nature furnishes a series of illustrations of the law of vital action in its *working by turns* in different parts of the same organism—an alternate working and rest. And the suspension of the more active working is not the result of disease, or any abnormal condition, but is in perfect harmony with a law inherent in physical nature.

We have also in this illustration a confirmation of the fact above mentioned, that vitality is limited in the sense of not being adequate to conduct all the operations of development and growth at the same time, and thereby declares most fully the great physiological law of alternation in the developmental processes of distinct parts toward a complete whole. If we turn now to the animal kingdom, we find the same law.

Dalton, in his physiology (p. 638) in presenting the subject of the development of the *cerebro spinal axis* gives an illustration of a fœtal pig five-eights of an inch long, showing the embryonic development of the brain and spinal cord. He remarks concerning the illustration thus: "It will be observed that the relative size of the various parts of the encephalon is very different from that which they afterwards attain in the adult condition. The cerebellum is very much inferior in size to the medulla oblongata. Soon afterwards the relative position and size of the parts begin to alter. The hemispheres and tubercula quadrigemina grow faster than the posterior portions of the encephalon. * * The relative dimensions of the parts are constantly changing." Let it be borne in mind that the different parts of the *cerebro spinal axis* begin their development at nearly the same time, but under the operation of the law of alternate vital action, the successive stages of development are unequal.

In the human subject the younger the embryo the larger are the head and upper parts in proportion to the rest of the body, showing the ascendency of one part over another in the rapidity of growth at different periods of development.

We read again in Dalton (p. 646) that "after the small intestine is once formed, it increases rapidly in length. It grows indeed faster than the wall of the abdomen, so that it can no longer be contained in the abdominal cavity, but protrudes in the form of an intestinal loop, or hernia, from the umbilical opening. This protrusion of the intestine can be seen during the latter part of the second month. At a subsequent period however the walls of the abdomen grow more rapidly than the intestine. They accordingly gradually envelope the intestine and at last enclose it again in the cavity of the abdomen." Viewing the embryonic development as here presented, the alternation of vital action is clearly manifest: and it will be readily seen that should anything occur to disturb this alternation at the proper time, the result would be a case of congenital hernia.

In dental science we have examples as fully illustrative of the law of *alternate vital action* as those already referred to. Tooth development can be studied intelligently only in connection with the development of the maxillary bones. The teeth have their after-growth, and life-long support in these bones. While zoologically and æsthetically speaking they may give character to the features of the face, physiologically the maxillary bones have their existence and development for, and in subservience, to the teeth as functional organs.

Along the semi-cartilaginous line of the embryo maxilla is seen as early as the sixth week of gestation a groove, and in this groove, a little later are seen two or more papilla. From the tenth to twelfth week the groove deepens, and membranous tongues are projected from the opposite sides at regular distances to form sacks for the tooth germs, and sockets for the future teeth.

No sooner is this condition assumed than the tooth papilla makes a rapid growth high above margins of the crypt in which it was sunken. Then for a few weeks it ceases development, and the surrounding sack takes its turn of growth, the papilla is again found below the surrounding membrane, the borders of which contract and shut the germ in. Thus the tooth germ alternates in growth with the sack which encloses it.

Pursuing the development farther we find the pulpy tooth appropriating lime salts in the process of dentification, and alternating this process with the ossification of the alveolus. Both processes do not proceed equally during the same period of time. It might be said that there is possibly a deficiency of bone phosphates, so that it would not be vitally economical to conduct the building up of both tooth and socket at the same time—and that a supply of more material, might set *the whole* machinery in motion. This statement assames that the strength of vital action exhibited, depends upon the amount of constituent elements artificially supplied.

But observing the operation of the law of development under the power of both vegetable and animal life, whether it be the development of organs near to or remote from each other, composed of the same or of different elementary constituents, all in harmony with the law of alternate vital action, it will be difficult to compel the assent of the judgment to any interpretation of natural processes which ignores that limit of vital action which induces alternation as an inherent law of organic function.

In tracing the operation of the law still further in the development of the dental organs, we find that when the imprisoned tooth is ready to emerge, with only its crown and neck dentified, vital development leaves here the incomplete root with only a sufficient amount of vital force to preserve the tissue *in tact* while the main force of developmental energy is employed in the emargination of the alveolus to let thc imprisoned tooth through. The work is now not building up of bony tissue, but tearing it down by absorption. Just so soon as this bony covering of the alveolus is removed the alternating work of tooth development again goes on with vigor, and the pearly crown emerges from the gum. When the crown is fairly through, vital action returns to the work of building up the walls of the alveolus to embrace firmly the neck of the tooth.

Having thus, by numerous examples, made evident the fact of limited vital force, and the law of its alternate action, I proceed to some observations of a practical nature, and to adduce some peculier conditions, poth of a physiological and pathological character, which this law may aid us in explaining.

Let it be borne in mind that all parts of the human body do not begin their development at the same time; that new functions are developed at different periods after birth; some organs are of slow growth, requiring many years to develop the full power of their functions, while others attain their complete functional power in a few years; that some organs after complete activity for a series of years, cease their function entirely. The age of twelve to sixteen years marks the period when the organs of reproduction are developed, and forty-five or near that, the period when the function ceases. It it is said that some of the carpel bones do not begin their ossification till twelve or fifteen years after birth. On the the other hand the teeth attain their full size and full performance of function at from twelve to fourteen years of age.

The teeth it will be observed are an example of early and rapid development. To accomplish this the vital force is taxed to a greater degree than is required for these organs in any after period in life. This greater activity of the vital force in developing the teeth implies a less activity somewhere else, or a cessation of the developmental process in some other organs Besides the extra amount of vital energy required at this period for the development of the teeth, another organ, the stomach, is undergoing a rapid development at the same time, and also takes from the sum of vital force with which the body is endowed a very considerable share. The stomach is undergoing a change in preparation for the new order of things to be introduced and made necessary by the function of the teeth. The stomach of the toothless infant is in no proper sense a stomach at all. It is little less than an intestinal enlargement. The right extremity of that portion which afterwards becomes a stomach passes so gradually into the intestine that the pilorus does not distinctly form the line of demarkation between the two. But when by the development of the teeth nature indicates the arrival of the time for nourishing the system with more solid food than milk, the vital forces are called to producing an organic change in the digestive system, Milk having been the only food of the infant, a kind of ailment which need no detention in the digestive tract to liquefy it, such an organ of digestion as is the stomach of an adult was not needed. But at the time of the emergence of the teeth the stomach assumes the form of a well distended pouch where solid food, too often poorly masticated, is detained and liquefied. This change of form requires a more than ordinary amount of the vital force·

This is the period of teething so fraught with disease and death to multitudes of the human family. Let us apply the law of alternate vital action, in explanation.

If the theory be correct that vitality is a limited and fixed sum, what is required extra of vital force to carry on the development of the teeth and the stomach must be withdrawn from some other organs, which will, for the time being, be feebly supported and therefore predisposed to functional disturbance from outward impressions. The lungs not being vitally supported as usual, pulmonary diseases may at this period be induced by very slight exposures. The skin also having contributed of its full vigor of vitality to the demand of the teeth and stomach, does not as fully void its its glands of the effete matter, and eruptions on the face and scalp appear. Another change made necessary by the development of teeth and a new diet, is the lengthening of the alimentary canal. It is a well-known fact that the relative length of the small intestine is not the same in the infant as in the adult. This work being in progress during the period of teething is an additional tax on the vital energy, which must, for the time leave the function of digestion feebly supported, and derangement of the bowels is a common result. The same susceptibility to functional disturbance may be noticed in other organs.

In tables of mortality we are furnished with a list of deaths by "teething," as though *teething* were a disease! We might as well call child-bearing a disease. It is true that the period of the deciduous teeth, for reasons set forth in this essay, is peculiarly open to sudden, fearful and fatal disease. But let us not confound the natural physiological process of teething, with the fearful train of diseases that sometimes attend it.

Let us pass on now to the age of puberty. At this period in life some of the most important organs of the whole physical economy pass to rapid maturity, viz: the organs of reproduction. The bodily languor, the listlessness, inattention, and general waywardness so often observable from the age of twelve to sixteen can no doubt be traced to a series of functional disturbances in organs weakened for the time being, in consequence of the extra amount of nervous vital energy required to devlop into maturity the functions of generation.

Observe now the limitation of vital force in the daily performance of bodily functions. With the stomach full and in the active process of digestion, the brain acts feebly and vigorous thinking is an impossibility. On the other hand while vigorous thinking occupies the brain, the operations of the stomach are disturbed. This is explained on the theory of limited vitality and the consequent necessity of *alternate action.* It is therefore unphysiological to require full-force operation at the same time, of both stomach and brain. But alternate functional action brings them into harmony with the law of *alternate vital action* and their working is thus made healthful.

I wish now to call your attention to the prevalence of dental diseases with women during pregnancy. If a woman in this condition be in health, all the functions of nature are in their most active state, each contributing its share to the production of like parts, and a symmetrical whole, of a new being in embryo. This being nature's chief, highest, and grandest work, she makes peremptory demand upon the system for all available energy even at a risk of less important organs and functions. Parts previously weakened by disease are now less able to resist the irritating and disturbing causes, and pain and distress follow. This is emphatically true of the teeth. Wisdom points to the remedy. Before this period arrives, the teeth should be put in the best possible condition of which dental skill is capable.

Let us consider for a moment toothache in the after part of the day. No man's vocation in life is carried on without a waste of bodily tissue, which the various functions of nature must daily reproduce. The use of the feet and hands, the movements of the body, the turning of the head, the act of seeing, hearing, tasting, smelling, the performance of internal bodily functions, the action of the brain for even a moment's thought, the expression of sentiment or emotion, all, and every one of these various phenomena are attended with a waste of organized substance which the bodily functions must daily reorganize. This performance of function is carried on by the nervo-vital force, an exciting cause. Waste and weariness are convertible terms in this connection. If there has been great waste, there is great weariness. vital forces are at once occupied for the recuperation of this lost tissue and strength for another day's labor. The diseased parts, in the meantime, are less vitally supported and declare the condition in which exhausted nature has left them, by the sensation of pain. If the teeth are rendered vulnerable by extensive decay, an *evening attack* of dentalgia may be confidently expected during the period for recuperation even to the extent of banishing sleep, "tired nature's sweet, sweet restorer."

It will have been observed in considering the subject thus far that alternate vital action implies an *alternate suspension* of vital action. It will therefore readily be seen that temporary reductions or suspensions of vital action in different parts of the body are not necessarily referable to pathological conditions, but are in perfect harmony with physiological law.

This leads me in closing this essay to allude to the grooved and pitted condition of the enamel not unfrequently seen in healthy mouths.

I know of no other account having been given of this condition of the teeth than that it is caused by a suspension of vital action in the enamel organ during the period of tooth development, prior to the emergence of the teeth from the gum, and induced by some constitutional disease, like measles or small pox. In view of the subject as it is here presented it is perfectly evident that there *might occur a suspension of vital action* in the enamel organ caused by the presence of such diseases as have been named, and others of like distinctive character. But it is not by any means evident to my mind that this grooved and defective condition is the result of such suspension of vital action. If the teaching of this essay be true, alternate activity and suspension of activity are the *law* of vital economy. This suspension does not, therefore, result in defect of tissue, but the functional work is resumed where it was left off, and carried on precisely as though it had not been suspended at all. This is according to nature, animate and inanimate, in all her developments.

There are reasons which to my mind militate against the aetiological account of this form of defective structure as commonly given in the text books of dentistry. We find that in the development of very many and probably all of the tissues of the body there are repeated suspensions of vital action, and yet the tissues become complete and perfect. It seems very strange, if true, that suspended vital action should be so peculiarly marked on the teeth, and not elsewhere in the bony structure; and strange indeed that it should not appear in the roots of teeth as well as in the crowns—roots that were in the in the dentifying process at the same time. There has never come under my notice a single case where like effects were seen on the roots of teeth. Furthermore, in my experience in operating on such teeth I have failed to find corresponding grooves in the underlying dentine. It appears therefore to be confined wholly to the enamel.

When I have been inquired of by patients and by the parents of children as to the cause of the grooved and pitted condition of their teeth, and I have named some of the constitutional diseases common to infants as the probable cause, I have very often been met by the remark that their children never had any of the diseases named, and had never been seriously ill. Of course my theorizing went to the ground in the face of such statements, and I was nonplused. In offering a new explanation of that condition of the teeth now under consideration, I dare not be dogmatical or very confident until by carefully conducted observation I am enabled to place my theory upon the more substantial basis of fact.

Premising thus, I am prepared to state my conclusions from only very limited observations, viz: that the horizontal grooving and pitting in the enemal of teeth is not the result of suspended vital action during the developmental process while in in the sacular stage, but is accomplished after the teeth have cut the gum, by a solvent; and that each groove and alternate ridge, mark successive stages of their emergence under the the law of alternate vital action.

July-2

It is well known that a very acrid fluid is poured forth from the margins of the gum when the mucus membrane is diseased, and that this very acrid secretion of the mucus follicles has physical characteristics which retain it in contact with the fresh crowns of the emerging teeth for a very considerable length of time, despite the washing with the more fluid saliva. We have seen in the earlier consideration of this subject, that the teeth do not make a uniform and steady growth and emergence from the gum, but that a growth of one, two or three months, is followed by a cessation of growth for a similar period of time. During this period when the teeth cease to grow, should they be constantly bathed along the line of contact with the margin of the gum, with the acrid fluid above mentioned, there must inevitably occur such a solution of the enamel as to form a furrow or groove. During the succeeding months of outward growth from the gum, the solvent is not long enough in contact with the enamel to produce results as in the preceeding months, and the enamel not wasted away is left in the form of a ridge. This process going on during the whole, or any considerable portion of the period of the emergence of the teeth, would produce a series of alternate ridges and grooves, and in a less dense structure of the enemal, lines of indentations or irregular depressions.

With a brief recapitulation I will close. I have called your attention to numerous physiological and pathological facts, some of which have been ascribed to disease, others to a freak of nature, and others still to constitutional idiosyncrasy or to accidental circumstances, which I have attempted to explain as occurring under the operation of a physiological law common to all vital organizations. If I have succeeded in establishing the fact of the existence of such a law, and made clear the explanation of the facts alluded to, and made possible a like explanation of an indefinite number of facts of physiology and pathology, three important branches of a noble science,—aetiology, diagnosis and therapeutics,—will have been made richer thereby, and mankind whether drawn by fortuitous circumtance or forced by necessity into suffering, will reap the benefit of applied science through the wisdom and skill of the kindred professions of medicine and dentistry.

